# Abiraterone acetate plus Prednisone/Prednisolone compared with Enzalutamide in metastatic castration resistant prostate cancer before or after chemotherapy: A retrospective study of real‐world data (ACES)

**DOI:** 10.1002/bco2.11

**Published:** 2020-03-27

**Authors:** Prantik Das, Sarah Taylor, James Price, Michael Jones, Cristina Martin‐Fernandez, Akram Ali, Thangrarajh Mugunthan, Chandrani Mallik, Colin Ward

**Affiliations:** ^1^ University Hospitals of Derby NHS Trust Derby UK; ^2^ School of Medicine University of Nottingham Derby UK; ^3^ Janssen-Cilag Limited High Wycombe UK

**Keywords:** abiraterone, castrate resistant, Enzalutamide, overall survival, progression free survival, prostate cancer

## Abstract

**Background:**

Abiraterone acetate combined with Prednisone/Prednisolone (AA+P) and Enzalutamide (ENZ) have proven survival benefit in men with metastatic castration‐resistant prostate cancer (mCRPC) in chemotherapy‐naïve and prior chemotherapy patients. There have been no studies directly comparing the effectiveness of ENZ to AA+P in mCRPC patients.

**Methods:**

A retrospective, survival analysis study of 143 real‐world mCRPC patients (90 in AA+P and 53 in ENZ group) was conducted. Patients who started their treatment between February 2012 and May 2016 were included. The primary end point was biochemical progression‐free survival (bPFS). Secondary end points were radiological progression‐free survival (rPFS) and overall survival (OS). Toxicity data were also collected. Data were analyzed using Cox proportional hazards (PH) models, adjusting for covariates: prior radical treatment; Gleason score; prostate‐specific antigen; age; and chemotherapy naïve or not.

**Results:**

After median follow‐up of 15 months (interquartile range 7 to 23), 112 events of biochemical progression were observed (71 in AA+P and 41 in ENZ). About 41% in AA+P group and 30% patients in ENZ group received prior chemotherapy. The chance of biochemical progression was significantly lower among ENZ patients than AA+P patients, when adjusting for all covariates in the Cox PH model (hazard ratio [HR] 0.54, 95% confidence interval [CI] 0.35 to 0.82, *P* = .004). There was a trend implying the chance of rPFS could be higher among ENZ patients than AA+P patients (HR 1.24, 95% CI 0.76 to 2.02, *P* = .4). There is no difference in OS between ENZ and AA+P patients, when adjusting for all covariates in the Cox PH model (HR 0.91, 95% CI 0.59 to 1.41, *P* = .7). About 38% of ENZ patients reported fatigue compared to 16% of AA+P patients, while hypertension was reported slightly more in AA+P patients.

**Conclusions:**

This study showed a statistically significant difference in bPFS, favoring ENZ, but no significant difference in rPFS or OS.

## 1 INTRODUCTION

Prostate cancer is the most commonly diagnosed cancer and the sixth leading cause of cancer‐related death among men worldwide.^1^ Advances in endocrine therapies have improved survival in men with high‐risk locoregional prostate cancer. However, new hormonal agents have been shown to extend survival in men with metastatic castration‐resistant disease.^1^, ^2^, ^3^ In most patients who are treated for advanced recurrent prostate cancer with androgen‐deprivation therapy (comprising a luteinizing hormone‐releasing hormone analogue or orchiectomy with or without an antiandrogen), disease progression occurs despite effective suppression of serum testosterone.^4^ This disease state, called castration‐resistant prostate cancer, is almost always associated with increases in levels of serum prostate‐specific antigen (PSA), suggesting that the disease continues to be driven by androgen‐receptor signaling.^5^

Abiraterone acetate (AA), a selective inhibitor of androgen biosynthesis, and Enzalutamide (ENZ), an androgen‐receptor–signaling inhibitor, have proven survival benefit in men with metastatic castration‐resistant prostate cancer (mCRPC) in chemotherapy‐naïve and after treatment with cytotoxic therapy.^1^, ^3^, ^6^, ^7^, ^8^

ENZ (MDV3100) is an oral androgen‐receptor blocker that binds more tightly and has a novel mechanism of action compared to older antiandrogens.^25^ The phase 3 AFFIRM trial^9^ compared ENZ to placebo in patients who progressed after cytotoxic chemotherapy, potentially with Prednisone or other glucocorticoids, and demonstrated superiority in all outcomes, including overall survival (OS), time to PSA progression, radiological progression‐free survival (rPFS), and PSA response rate.^24^

The multinational, double‐blind, randomized, placebo‐controlled, and phase III PREVAIL study^6^ evaluated ENZ in men with chemotherapy‐naïve mCRPC that had progressed despite the use of androgen deprivation therapy and showed significantly decreased risk of radiological progression and death and delayed time to initiation of chemotherapy.

The phase 3 COU‐AA‐301 trial^1^ compared Abiraterone Acetate plus Prednisone (AA+P) versus placebo plus Prednisone and demonstrated superiority in all outcomes, including OS, time to PSA progression, rPFS, and PSA response rate.

In the chemotherapy‐naïve setting, phase 3 COU‐AA‐ 302 trial^3^, ^7^ with a median follow‐up of more than 4 years has demonstrated that treatment with AA+P prolonged OS compared with placebo plus Prednisone by a margin that was both clinically and statistically significant.

So far there is no phase 3 data directly comparing the effectiveness of ENZ to AA+P in chemotherapy‐naïve mCRPC or after treatment with Docetaxel. This study is aiming to evaluate the efficacy of AA+P and ENZ in men with mCRPC who demonstrated disease progression radiologically or biochemically before or after Docetaxel chemotherapy.

## 2 METHODS

### 2.1 Patients

Patients were eligible to be included in the dataset if they had mCRPC, began treatment with either AA+P or ENZ between February 2012 and May 2016 (inclusive), but had not received treatment with both AA+P and ENZ during this time period, and were at least 18 years old when starting AA+P or ENZ treatment.

The patients were identified from our institutional data bases. The data were collected from electronic and paper case notes, electronic chemotherapy prescribing system (Chemocare), nursing records, pathological data base and accessing radiological reports, and imaging through Picture Archiving and Communication System.

### 2.2 Study design and treatment

This is a retrospective, single center, survival analysis study, using real‐world data from patients with mCRPC, split into cohorts for comparative analyses by treatment pathways. Patients were categorized to the following cohorts: Patients given AA+P; and patients given ENZ. Routine data on treatments given and measurements taken was collected for each participant over a 24 months period, starting with the patient receiving AA+P or ENZ for the first time. Due to the retrospective nature of this study, there was no randomization or blinding.

The primary end point was biochemical progression‐free survival (bPFS) at 12 months, defined as the time from starting AA+P or ENZ to an event of biochemical progression (a 25% increase in PSA over the nadir and an absolute increase in PSA by at least 5 ng per milliliter, confirmed by a second value).

The secondary end points included OS, defined as the time from starting AA+P or ENZ to death from any cause, and radiological PFS, defined as the time from starting AA+P or ENZ to an event of radiological progress (according to modified Response Evaluation Criteria in Solid Tumors^10^). Toxicity data were also collected as a secondary end point.

### 2.3 Study assessment

A number of routine measurements were also collected to be used in descriptive baseline assessments, as covariates, and as subgroup identifiers to the primary and secondary analyses; These included: PSA at diagnosis, baseline, final visit, and lowest value from intermediate visits; Gleason score at diagnosis; T stage at diagnosis; N stage at diagnosis; M stage at diagnosis; treatment history throughout; performance status at baseline; previous chemotherapy/chemotherapy naive; number of AA+P or ENZ cycles; reason for stopping AA+P or ENZ treatment; and toxicity.

### 2.4 Study oversight

The study was designed by the authors, then reviewed and approved by employees of the sponsor, institution and employees of the funder, Janssen‐Cilag Ltd. The analyses of the data were performed by a statistician employed by the sponsor, and the results were reviewed by all of the authors, employees of the sponsor, and employees of the funder. The first draft of the manuscript was written by some of the authors, then reviewed and approved by the other authors, the sponsor, and the funder.

### 2.5 Statistical analysis

The average PFS rate at 12 months across both treatment groups in the data collected was 21.6%. The minimal clinically important constant hazard ratio (HR) was calculated using this observed average PFS rate and the minimum number of participants in any group already collected, 50.

When the sample size in each group is 50, with a total number of events required, E, of 31, a.050 level two‐sided log‐rank test for equality of survival curves has 80% power to detect the difference between a AA+P group proportion *π*1 at 12 months of 0.216 and a ENZ group proportion *π*2 at 12 months of 0.476 (a constant HR of 2.064).

Cox proportional‐hazards (PH) model was used for the primary analysis of survival data for comparison of treatment groups. The Cox PH assumptions have been tested and found valid. Log‐rank tests for equality and Kaplan‐Meier plots were also performed as secondary analyses of survival data for comparison of treatment groups. Fisher's exact test was used to compare decrease in PSA between treatment groups and descriptive percentages were used for the toxicity data.

## 3 RESULTS

### 3.1 Patients and treatments

**FIGURE 1:**
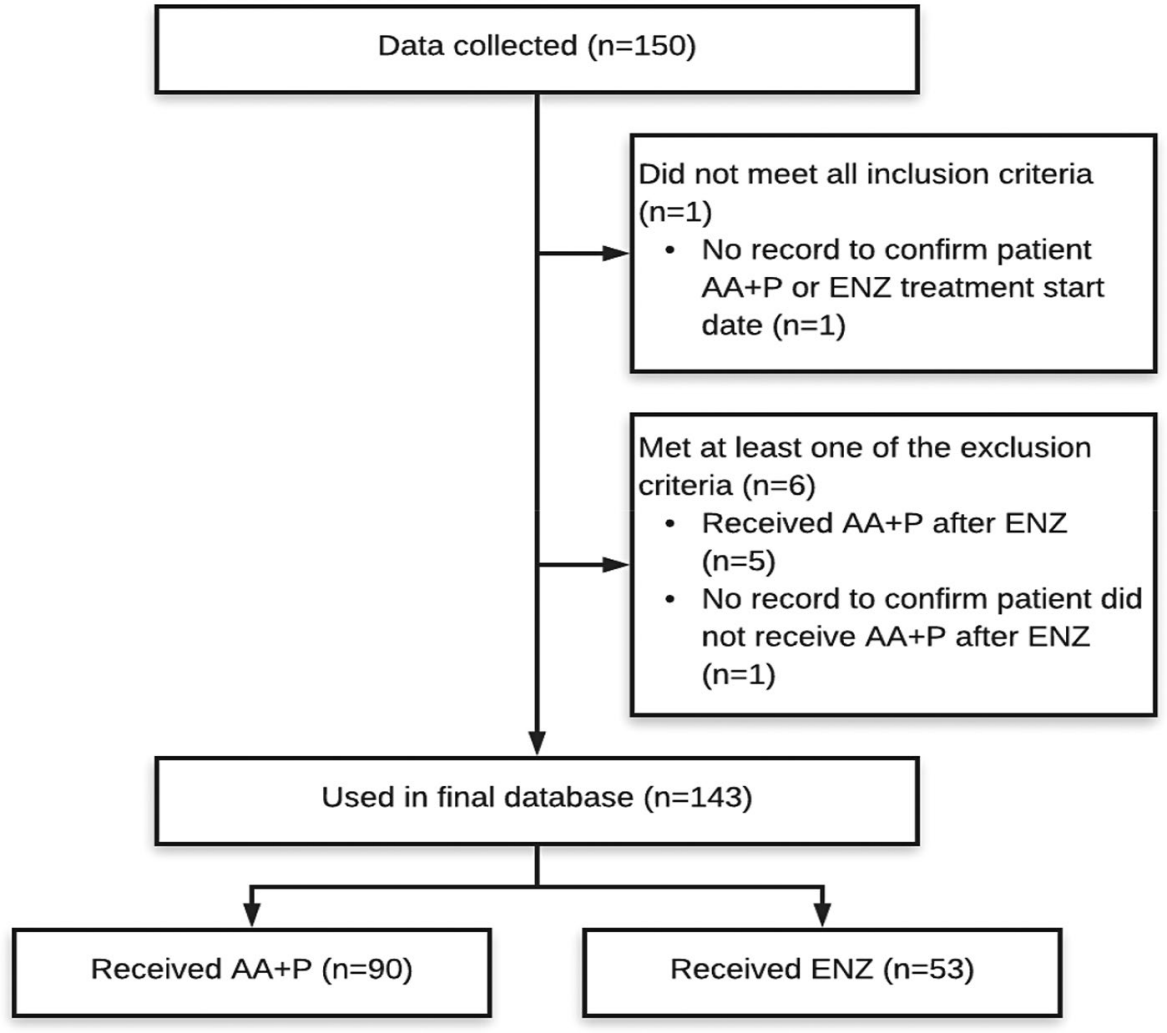
Patient flowchart. AA+P, abiraterone acetate plus prednisolone; ENZ, enzalutamide

Data were collected on 143 eligible patients, of which 90 had received AA+P and 53 had received ENZ (Figure 1). The majority of the baseline demographics were shown to be evenly distributed across the two treatment cohorts (Table 1). The only exception to this was the start date of AA+P/ ENZ treatment, for which the AA+P cohort's median start date (November 2013; interquartile range (IQR), June 2013 to July 2014) was over a year earlier than the ENZ cohort's median start date (December 2014; IQR, October 2014 to July 2015). It is worth highlighting alongside this that the proportion of patients whose follow‐up was cut off by the study end date for data collection was not significantly different (*P* = .4) between the AA+P cohort (18%) and the ENZ cohort (25%). Within both treatment cohorts, most patients had not received chemotherapy prior to starting AA+P or ENZ treatment (AA+P, 59%; ENZ 70%).

**TABLE 1:** Baseline demographic and clinical characteristics of the patients

Characteristic	AA+P (n = 90)	ENZ (n = 53)	*P* value
Baseline demographics			
Age	75 (73 to 76)	74 (71 to 77)	.9
*mean (95% CI)*	*(n = 90)*	*(n = 53)*	
PSA at diagnosis	79 (21 to 276)	61 (29 to 310)	.9
*median (IQR)*	*(n = 78)*	*(n = 52)*	
Start date of AA+P/ENZ treatment	November 10, 2013 (June 25, 2013‐July 10, 2014)	December 17, 2014 (October 1, 2014‐July 18, 2015)	<.00005
*median (IQR)*	*(n = 90)*	*(n = 53)*	
Patients censored by data collection cutoff *n (%)*	16 (18)	13 (25)	.4
Gleason score *n (%)*			.6
6	6 (12)	2 (5.3)	
7	10 (19)	10 (26)	
8	7 (13)	3 (7.9)	
9	27 (52)	20 (53)	
10	2 (3.9)	3 (7.9)	
Performance status at baseline *n (%)*			1.0
0‐1	71 (91.0)	46 (90.2)	
+2	7 (9.0)	5 (9.8)	
T stage at diagnosis *n (%)*			.9
1	1 (1.1)	0 (0)	
2	2 (2.2)	3 (5.66)	
3	8 (8.8)	5 (9.43)	
4	1 (1.1)	0 (0)	
Not available	78(86.8)	45(84.9)	
N stage at diagnosis *n (%)*			.5
0	9 (82)	8 (100)	
1	2 (18)	0 (0)	
Not available			
M stage at diagnosis *n (%)*			
0	12 (14)	8(18)	
1	78 (86)	45(82)	
Radiotherapy or surgery in the radical setting *n (%)*			.5
RT	10 (12)	7 (13)	
Surgery	0 (0)	1 (1.9)	
No radical treatment	76 (88)	45 (85)	
Received docetaxel (prior to AA+P/ENZ) *n (%)*			.2
No (pre‐chemotherapy)	53 (59)	37 (70)	
Yes (post‐chemotherapy)	37 (41)	16 (30)	
Follow up	15 (7 to 23)	14 (8 to 30)	.9
*median (IQR)*	*(n = 90)*	*(n = 53)*	
Time to progression (months)	1.7 (0.9 to 5.3)	6.5 (2.6 to 11)	<.0001
*median (IQR)*	*(n = 90)*	*(n = 53)*	

Abbreviations: AA+P, abiraterone acetate plus prednisolone; CI, confidence interval, ENZ, enzalutamide; PSA, prostate‐specific antigen.

### 3.2 Efficacy

Treatment with AA+P resulted in a 46% increase in the risk of biochemical progression when compared to ENZ (HR, 0.54; 95% confidence interval [CI], 0.35 to 0.82; *P* = .004), adjusting for all full model variables: Radical treatment; Gleason score; PSA; age; and prior Docetaxel. A total of 112 patients had a recorded event of biochemical progression: 71 patients in the AA+P cohort (79%) with a median bPFS of 5.1 months and 41 patients in the ENZ cohort (77%) with a median bPFS of 8.9 months.

**FIGURE 2:**
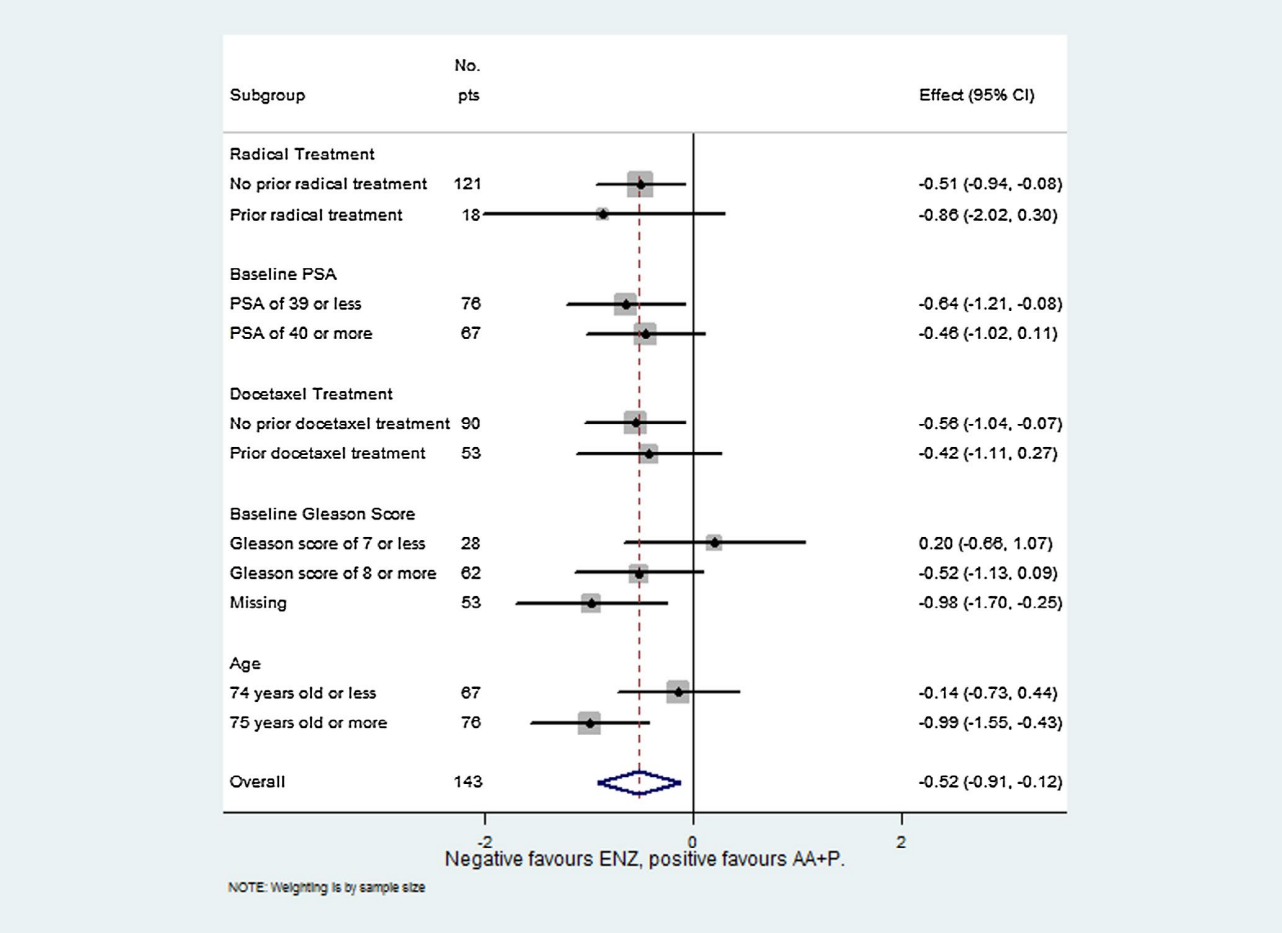
Hazard ratios for the risk of biochemical progression, according to subgroup sub groups forest plot_20191016

Univariate analyses conducted on the subgroup of each variable used in the primary analysis full model showed a significant difference in bPFS between the two treatments, in favor of ENZ, for the following subgroups: Prior radical treatment; baseline PSA of 39 or less; no prior Docetaxel; baseline Gleason score missing; and age 75 years or older (Figure 2).

Within the subgroup of patients who received no Docetaxel prior to AA+P/ENZ, treatment with AA+P resulted in a 44% increase in the risk of biochemical progression when compared to ENZ (HR, 0.56; 95% CI, 0.33 to 0.94; *P* = .03), adjusting for all full model variables. Within the subgroup of patients who received Docetaxel prior to AA+P/ ENZ, there is no statistical significant difference in biochemical progression between two treatment groups (HR, 0.49; 95% CI, 0.23 to 1.08; *P* = .08), adjusting for all full model variables.

Treatment with AA+P resulted in a 9% increase in the risk of death (HR, 0.91; 95% CI, 0.59 to 1.41; *P* = .7) when compared to ENZ, adjusting for all full model variables. A total of 101 patients died: 68 patients in the AA+P cohort (76%) with a median OS of 17 months and 33 patients in the ENZ cohort (62%) with a median OS of 19 months.

No significant difference between treatments was found in OS for both patients who received no Docetaxel prior to AA+P/ENZ treatment subgroup (HR, 0.81; 95% CI, 0.44 to 1.48; *P* = .5) and patients who received Docetaxel prior to AA+P/ENZ treatment subgroup (HR, 1.13; 95% CI, 0.58 to 2.18; *P* = .7).

Treatment with AA+P resulted in a 24% decrease in the risk of radiological progression numerically (HR, 1.24; 95% CI, 0.76 to 2.02; *P* = .4) when compared to ENZ, adjusting for all full model variables. A total of 77 patients had a recorded event of radiological progression: 45 patients in the AA+P cohort (50%) with a median radiological PFS of 13 months and 32 patients in the ENZ cohort (62%) with a median rPFS of 12 months.

No significant difference between treatments was found in rPFS for both patients who received no Docetaxel prior to AA+P/ENZ treatment subgroup (HR, 1.01; 95% CI, 0.52 to 1.95; *P* = 1.0) and patients who received Docetaxel prior to AA+P/ENZ treatment subgroup (HR, 1.76; 95% CI, 0.77 to 4.03; *P* = .2).

### 3.3 Safety

**FIGURE 3:**
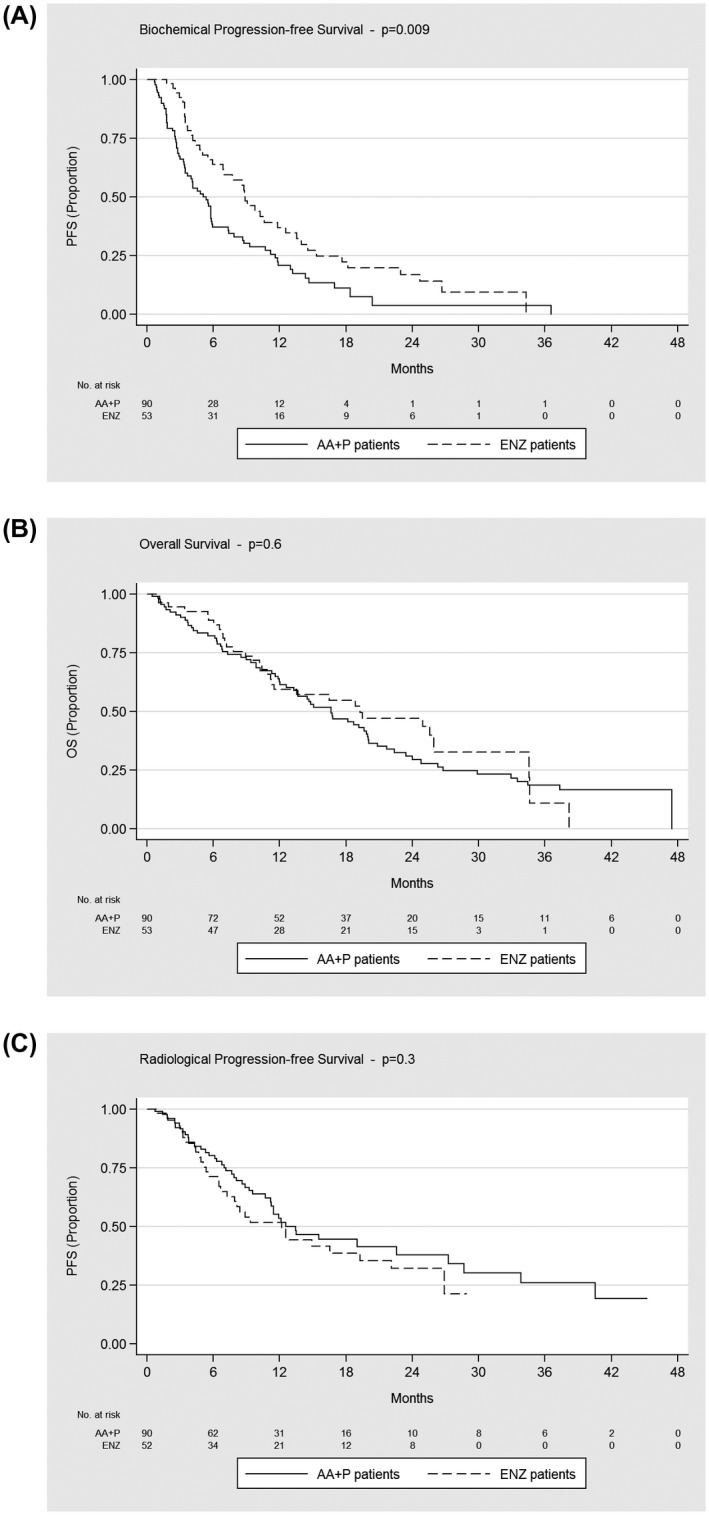
Kaplan‐Meier estimates of biochemical progression‐free survival, overall survival, and radiological progression‐free survival. AA+P, abiraterone acetate plus prednisolone; ENZ, enzalutamide; PFS, progression free survival; OS, overall survival

**FIGURE 4:**
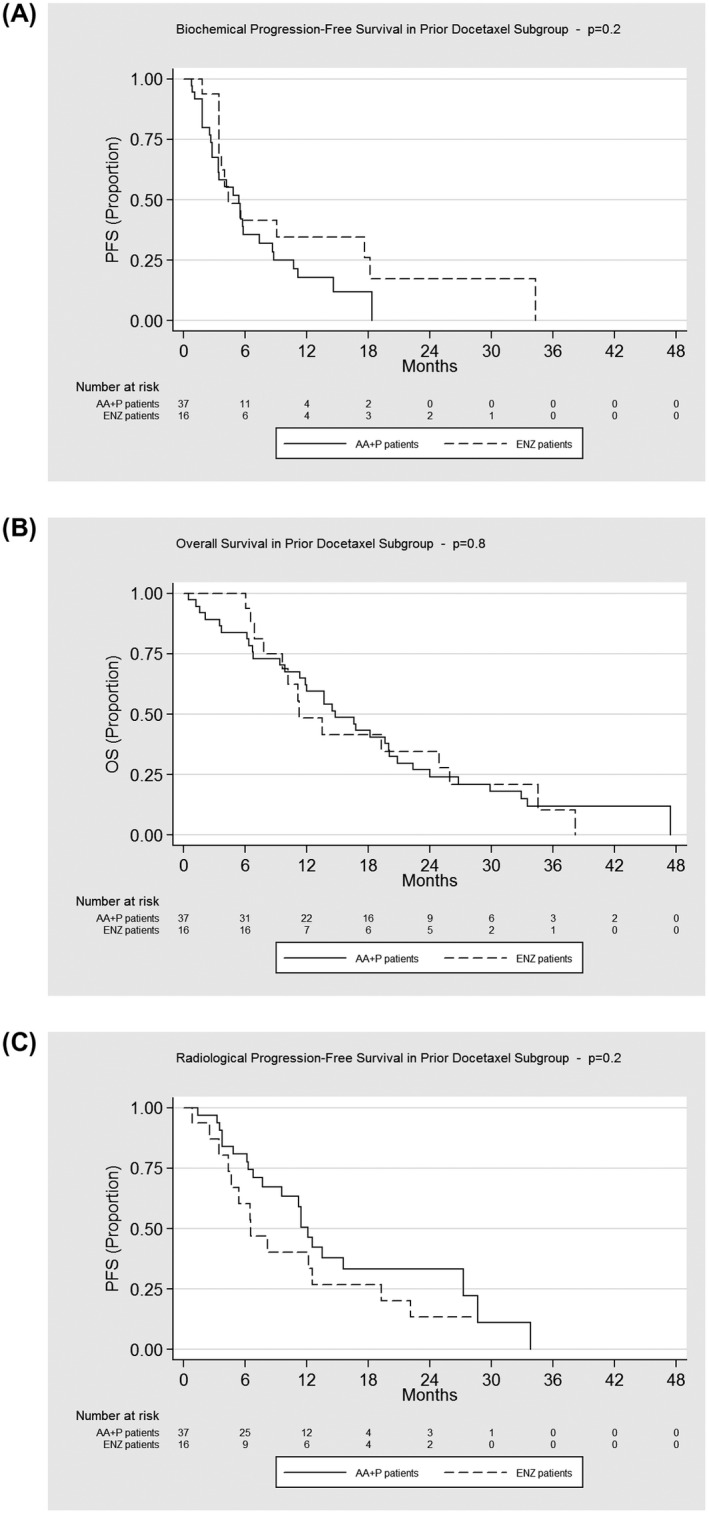
Kaplan‐Meier estimates of biochemical progression‐free survival, overall survival, and radiological progression‐free survival by prior docetaxel subgroups. AA+P, abiraterone acetate plus prednisolone; ENZ, enzalutamide; PFS, progression free survival; OS, overall survival

**FIGURE 5:**
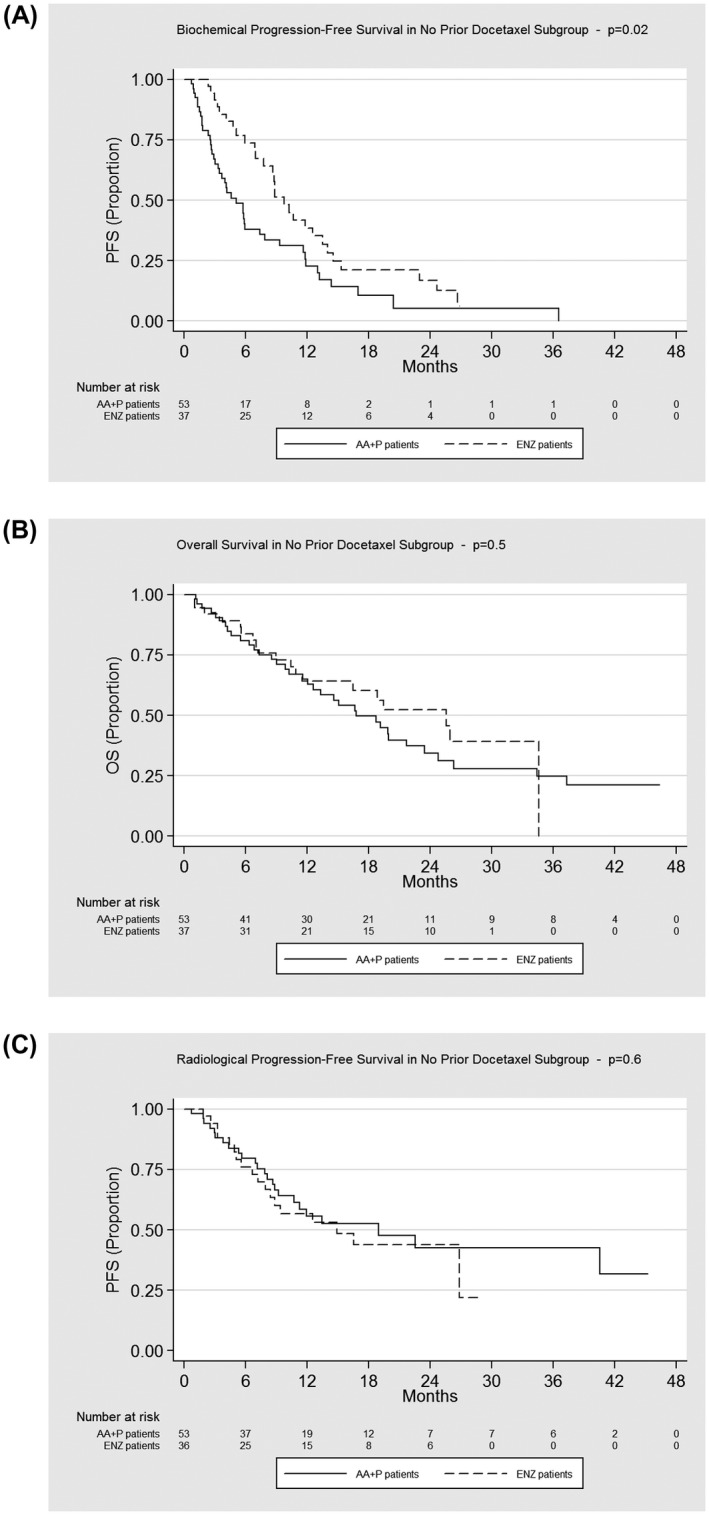
Kaplan‐Meier estimates of biochemical progression‐free survival, overall survival, and radiological progression‐free survival by no prior docetaxel subgroups. AA+P, abiraterone acetate plus prednisolone; ENZ, enzalutamide; PFS, progression free survival; OS, overall survival

Both drugs demonstrated a favorable safety profile overall (Table 2). Grade 3 or higher adverse events were reported in patients with both agents: Hypertension was more common among patients treated with AA+P (2.2% vs 0%), whereas patients treated with ENZ reported more dizziness than with AA+P (5.7% vs 2.2%), and a very small number of patients with ENZ reported confusion (1.9% vs 0%). Tiredness was significantly higher within the ENZ group than within the AA+P group (38% vs 16%); this is clinically important given the significant impact of lethargy on quality of life. Only 1% vs 0% of patients experienced grade 3 impairment of liver function tests with AA+P compared with patients treated with ENZ. Discontinuation rate was extremely low in both treatment groups (Figures 3, 4, 5).

**TABLE 2:** Toxicity results

Event	AA+P (n = 90)	ENZ (n = 53)
	n (%)	
Back pain	2 (2.2)	1 (1.9)
Confusion	0	1 (1.9)
Deterioration	0	1 (1.9)
Diarrhea	1 (1.1)	0
Dizziness	2 (2.2)	3 (5.7)
Fatigue	14 (16)	20 (38)
Fatigue grade 1	0	4 (7.5)
Fatigue grade 2	1 (1.1)	0
Fatigue grade 3	0	2 (3.8)
Falls	0	1 (1.9)
High alanine aminotransferase (22‐24)	1 (1.1)	0
Hot flushes	2 (2.2)	4 (7.5)
Hypertension	2 (2.2)	0
Mood swings	1 (1.1)	0
Nausea	2 (2.2)	0
Severe impairment of LFTs	1 (1.1)	0

## 4 DISCUSSION

Our retrospective study included patients with mCRPC who had or had not received Docetaxel chemotherapy before initiating either AA+P or ENZ.^1^, ^6^, ^7^, ^11^ Overall, the bPFS was longer for the patient group treated with ENZ over those treated with AA+P. When the data were analyzed for prior Docetaxel exposure or chemotherapy naivety, the statistical improvement in bPFS with ENZ was not maintained for the post‐Docetaxel groups. We feel that this may reflect the small subgroup numbers, in which despite a trend toward ENZ, was unable to prove statistical significance. In our experience, biochemical progression correlates closely with the overall clinical picture and patient symptoms, we, therefore, feel that that these results may be relevant when considering our patients in clinic.

The benefit of ENZ in the context of bPFS for the whole population was evident in some of the prespecified subgroup analyses, with the most marked difference seen in patients with no prior radical treatment, baseline PSA of 39 or less, no prior Docetaxel, baseline Gleason score missing, and age 75 years or older, respectively.

Our findings, therefore, suggest that the use of ENZ may be preferable in those patients with no prior radical treatment. It may suggest that there could be differences in the tumor biology of those with subsequent metastatic disease in comparison to upfront metastatic disease, which responds preferentially to ENZ. Although our study numbers are small and it is difficult to make definite conclusions, this generates an interesting hypothesis and potential area for future research.

There was no statistical difference in OS between the two treatment groups. It is possible that the OS data were impacted on by subsequent treatment lines received by patients following ENZ/AA for which we did not collect data, especially in light of known research showing patients pretreated with ENZ respond poorly to subsequent AA, whereas the reverse is not true.^12^ However, within our center it would be very unusual for patients to be treated with both agents given that this is not a NHS funded pathway currently.

Similarly, the study did not demonstrate any statistically significant difference in rPFS between the two treatment groups. In clinical practice, we tend to rely more upon biochemical and symptom response to therapy rather than ensuring regular radiological assessment; this may impact on the rPFS results given the frequency of interval scans would not have been as reliable as serial PSA assessments.

There were 29 reported grade 3 and above adverse events in the AA+P cohort (mean 0.32 events per patient) and 37 adverse events in the ENZ cohort (mean 0.70 events per patient). The most common events were fatigue (AA+P, 16%; ENZ, 38%) and hot flushes (AA+P, 2.2%; ENZ, 7.5%). Treatment with both agents were well tolerated any no new safety signal were identified. No patients required hospital admission due to toxicities.

## 5 APPLICATION WITHIN “REAL WORLD”

Our attempt is unique as it compared both AA+P and ENZ in a pre‐ and post‐chemotherapy setting within a single study.^1^, ^3^, ^6^, ^7^

Our primary end point was bPFS at 12 months after the start of treatment with AA+P or ENZ and we have demonstrated a favorable response with ENZ in terms of bPFS, although not radiological. In addition, we have not found a survival difference between the two agents. The study has some interesting prespecified subgroups, for example, previous radical treatment, which is an attempt to investigate whether patient's outcome differs if the disease started locally and then metastasized or metastatic disease from outset. Our findings indicated that patients with high‐risk features, such unknown Gleason score (with lack if biopsy suggesting high‐risk disease), responded better with ENZ.

A recent retrospective cohort study and data presented by Kalaf et al have suggested a superior PSA response rate with ENZ over AA within a pre‐chemotherapy setting, with a superior time to PSA and disease progression seen in patients over 80 years.^12^, ^13^, ^14^

In regular clinical practise, PSA response rate was a commonly used marker to evaluate efficacy for CRPC, although there is no conscious agreement.^15^ Study by Smit et al suggested a higher PSA response rate was associated with longer survival.^16^ Interestingly a meta‐analyses also found that ENZ had greater benefits in PFS but not in OS though number of study included in the analyses were small.^17^ A recently published systemic review has indicated ENZ group had a significantly higher PSA response rate.^18^

The study reflects outcomes with AA+P and ENZ within a real life population, which is therefore directly applicable to the clinic. The mean age of patients was 74 years in the ENZ group and 75 years in the AA+P group. It included patients with performance statuses of 0‐3, those without biopsies, patients who had received prior radical treatment and pre‐ or post‐chemotherapy. The heterogeneity of the population in this study is rarely seen within a randomized clinical trial.

In our study, the median bPFS for patients treated with AA+P was 5.1 months. (AA+P with no prior Docetaxel 5 months and with prior Docetaxel 5.4 months, respectively). Within the landmark COU‐AA‐301 study, the time to PSA progression with AA+P after prior Docetaxel was 10.2 months and within COU‐AA‐302 pre‐chemotherapy was 11.1 months.^3^, ^11^ For patients treated with ENZ, our overall bPFS was 8.9 months (ENZ with no prior Docetaxel 9.8 months and with prior Docetaxel 4.4 months, respectively). Within the PREVAIL trial of ENZ in chemotherapy‐naïve patients, the median bPFS was 11.2 months.^20^ The bPFSs that we demonstrated were significantly lower than those seen within these landmark trials.^1^, ^7^, ^11^, ^20^ It is likely that this reflects the efficacy of the drugs within an unselected clinic population, rather than a prespecified trial population. Also our overall bPFS for both agents are combined data for pre‐ and post‐chemotherapy setting, it is possible that which may influence the overall result.

This study showed the median OS for the ENZ treated patient population was 19 months vs 17 months for AA+P treated patients. Our post‐chemotherapy patients demonstrated OS of 15 months with AA+P and 11 months with ENZ, respectively (*P* = .8); These figures are more comparable to landmark trials in the post‐chemotherapy setting (COU‐AA‐301 14.8 months, AFFIRM 18.4 months).^11^, ^21^ For chemotherapy‐naïve patients in our study, OS is 26 months with ENZ and 17 months with AA+P (*P* = .5).

A previous study by Fang et al attempted to pool the HR based on median OS and PFS. This study showed better OS associated with ENZ in pre‐docetaxel group but there was no difference in OS in post‐chemotherapy setting. However, the authors explained, the estimated HR could bring considerable uncertainty and was weakly convincing.^22^

A systemic review by Zhang and colleagues, which aimed indirect comparison between AA and ENZ, has shown that ENZ outperformed abiraterone with respect to PSA PFS, rPFS, and PSA response rate. However, there was no significant difference with regard to OS.^23^ This result are consistent with our findings though our cohort did not demonstrate any difference in rPFS.

We included patients with a performance status up to 3, whereas within PREVAIL, the inclusion criteria was an ECOG performance status of 0‐1, with 67% of the patients having a performance status of 0. Although within COU‐AA‐301 they allowed patients with ECOG PS 0‐2 and 90% of the patients had a performance status of 0‐1, which was not dissimilar to our study, it is not specified how many patients had a PS of 0 within this group.^11^ We also included patients who were highly symptomatic when commencing the drug, whereas in PREVAIL, patients had to be a maximum of mildly symptomatic for inclusion, with 66.2% having 0‐1 pain scores out of 10.^6^, ^20^ These results are important as they may help to guide us when discussing treatment options in patients with a poorer performance status or who are highly symptomatic and ensure that we are being realistic in terms of the expected outcomes of the therapy.

## 6 STUDY LIMITATIONS

Given the retrospective nature of our study, there was no formal randomization between therapy groups; this has the potential to introduce bias through the physician's choice of agent and rationale. In our experience, primary factors for favoring ENZ over AA would be a background of liver disease or impaired liver function tests, poorly controlled hypertension, background of hypokalaemia or concerns associated with steroid use such as diabetes or heart failure. AA would be used in preference over ENZ where a background of preexisting marked lethargy or a history of previous seizure was present. Aside from these factors related to comorbidity, the treating clinicians within our center have varied practice between their preferred agents. The groups were not matched for baseline characteristics. Also, because the data were collected in a retrospective manner, the quality of documentation of adverse events was suboptimal at times and may impact on the accuracy of event reporting and grading. Not all patients had a histological diagnosis with Gleason grading, although through our knowledge of local practice, we are confident that this group represents those with very high PSA values and aggressively behaving disease. As previously, mentioned, not all patients underwent regular imaging which could have impacted on the assessment of rPFS.

Data were not analyzed regarding patients’ subsequent treatment lines following ENZ or AA. It is therefore possible that this influenced the OS data as above.

## 7 CONCLUSION

In conclusion, our real‐world comparison between ENZ and AA+P showed a statistically significant difference in bPFS favoring ENZ overall, which was most marked within the high‐risk patient groups. However, there was no statistical difference in OS or rPFS. Both agents are reasonably well tolerated. These findings could potentially guide treatment decision making, however, with caution given the retrospective non‐randomized nature of the study.
